# Changes in Global Gene Expression in Response to Chemical and Genetic Perturbation of Chromatin Structure

**DOI:** 10.1371/journal.pone.0020587

**Published:** 2011-06-03

**Authors:** Karen Hudson, Song Luo, Nicole Hagemann, Daphne Preuss

**Affiliations:** Howard Hughes Medical Institute, Department of Molecular Genetics and Cell Biology, University of Chicago, Chicago, Illinois, United States of America; Ludwig-Maximilians-Universität München, Germany

## Abstract

DNA methylation is important for controlling gene expression in all eukaryotes. Microarray analysis of mutant and chemically-treated *Arabidopsis thaliana* seedlings with reduced DNA methylation revealed an altered gene expression profile after treatment with the DNA methylation inhibitor 5-aza-2′ deoxycytidine (5-AC), which included the upregulation of expression of many transposable elements. DNA damage-response genes were also coordinately upregulated by 5-AC treatment. In the *ddm1* mutant, more specific changes in gene expression were observed, in particular for genes predicted to encode transposable elements in centromeric and pericentromeric locations. These results confirm that DDM1 has a very specific role in maintaining transcriptional silence of transposable elements, while chemical inhibitors of DNA methylation can affect gene expression at a global level.

## Introduction

DNA cytosine methylation, specifically at CG dinucleotides plays a role in maintaining gene silencing and in gene imprinting. In plants 5-methylcytosine can also occur in asymmetric contexts, such as CNG or CNN (where N can be any other nucleotide), as well as at CG sites. In *Arabidopsis*, CG methylation is maintained by the methyltransferase *MET1* [1], while *CHROMOMETHYLASE3* (*CMT3*) and the *DOMAINS REARRANGED METHYLASE*s (*DRM1* and *DRM2*) are involved in the maintenance of CNG and CNN methylation respectively [2], [3], [4], [5], [6]. In addition, plants exhibit strand-specific methylation of cytosines in pericentromeric regions [7]. DNA cytosine methylation is typically associated with transcriptional silence in plants [8]. Cytosine methylation has been shown to be required for maintaining transcriptional silence of transposable elements through an RNAi-dependent pathway, and has also been shown to be required for parent-of-origin specific expression of the *FWA* gene in *Arabidopsis* endosperm [9], [10].

*DDM1* (*Decrease in DNA Methylation1*) encodes a SWI2/SNF2-like chromatin remodeling factor that is required for normal genomic DNA methylation and transgene and transposon silencing [11]. The *ddm1* mutant was originally identified on the basis of hypomethylation of satellite repeats [12]. DDM1 is also required to maintain the normal pattern of histone modifications (H3mK9) at the chromosome IV heterochromatic knob [13]. *ddm1* mutants have been reported to have a wide array of morphological and physiological defects as a result of misregulation of a number of genes due to aberrant chromatin structure, including a delay in flowering arising from hypomethylation at the FWA locus [14], [15], [16], [17]. Some studies have demonstrated a requirement for cytosine methylation prior to the establishment of heterochromatic histone methylation marks, while other studies show that in some contexts, histone modification can occur without DNA methylation [18].

To determine to what extent gene expression in *Arabidopsis* is regulated by chromatin structure and DNA methylation state, microarray expression profiling was used in conjunction with a chemical treatment to perturb chromatin structure of wild-type *Arabidopsis* seedlings, as well as in the *ddm1* mutant background. The methylation inhibitor 5-aza-2′ deoxycytidine (5-AC) inhibits the mammalian Dnmt1 cytosine methyltransferase which is homologous to *Arabidopsis* MET1 [19] and this treatment has been shown to reactivate expression of silenced nucleolar genes in plants [20], [21]. Since *ddm1* was originally identified on the basis of altered methylation at centromere satellite repeats, and is known to have effects on chromatin structure specifically in the heterochromatic regions of the centromeres, we sought to compare the effect of loss of DDM1 on gene expression with the more general chromatin effects provided by treatment with a chemical inhibitor of methylation.

## Results

### Gene expression differences in the *ddm1* mutant and in response to chemical treatment

Reduction of DNA methylation in the samples was assessed by comparative chromatin immunoprecipitation of control and 5-AC-treated DNA with an anti-5-methylcytosine antibody, and by sequencing bisulfite-treated genomic DNA for control, 5-AC-treated, and *ddm1* seedlings. Bisulfite sequencing revealed an 80% reduction in asymmetric cytosine methylation after 5-AC treatment, and complete loss of asymmetric cytosine methylation in *ddm1* for a methylated region of the *CLAVATA2* gene examined. CNG methylation was completely lost after 5-AC treatment (Figure S1A). CG methylation was lost at 60% of CG sites in *ddm1*. A reduction in CG methylation was observed for 5-AC treatment on a per-sample basis, six CG sites in the sequenced region are methylated in 90–100% of control samples, these were found to be no more than 80% methylated after 5-AC treatment and no more than 30% methylated at any CG site in the *ddm1* plants. This is consistent with the method of induction of loss of methylation in the treated and mutant samples. The *ddm1* mutants have lost methylation over generations, while the chemical treatment results in loss of methylation in a subset of actively dividing cells. Additionally, efficiency of precipitation of methylated centromeric repeats was reduced by more than 50% after 5-AC treatment (Figure S1B). Together, these data suggest that methylation is reduced in the 5-AC-treated and *ddm1* samples with respect to the control.

Complete lists of genes showing differential expression in this study are provided in Tables S1, S2, S3. Table S1 lists 35 genes that were significantly differentially expressed in the *ddm1* mutant. While many genes on the array had high fold-change values, variation was also high (Figure S2); therefore a stringent false-discovery corrected cutoff was imposed to arrive at this list of genes specifically and reproducibly affected in the *ddm1* mutant (see Materials and Methods). These genes are upregulated up to 100-fold. Twenty-five of the *ddm1*-regulated genes were also found to be significantly up-regulated after 5-AC treatment (Table S2), but the remaining 10 genes were up-regulated less than 3-fold after 5-AC treatment. Tables S2 and S3 list the genes found to be over- and under-expressed after 5-AC treatment, respectively. These genes span all functional categories.

### Loss of DDM1 selectively affects transcription of transposable elements

**Figure 1:**
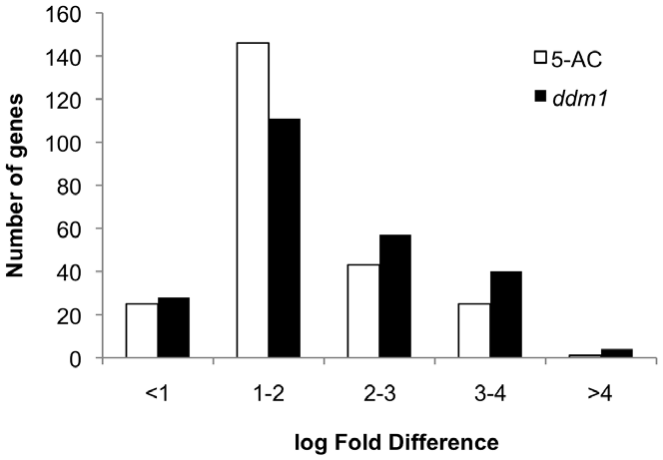
Transposable element genes are upregulated in the *ddm1* mutant and after 5-AC treatment. Log expression differences (5-AC/Ws or *ddm1*/Ws) for 240 probesets annotated as transposable elements that were present on the microarray.

Since it is known that both DNA methylation and DDM1 are involved in silencing transposable elements and pseudogenes, we examined the responses of these two classes of genes. Figure 1 shows the fold induction in response to 5-AC treatment or in the *ddm1* mutant for all of the genes (240) annotated by the Arabidopsis Genome Initiative (AGI, see Methods) as “transposable elements” and called “present” in this study by the MAS Version 5.0 software (Affymetrix). In both *ddm1* mutants and 5-AC treated seedlings, many transposable elements were upregulated by 100-fold or more. The Affymetrix ATH1 microarray contains probes for 113 genes that are annotated as “pseudogenes”. Of the 33 probes called present in this experiment annotated as pseudogenes, only two of these genes change significantly in expression in either the *ddm1* mutant or as a result of treatment with 5-AC (not shown). This is consistent with the role for cytosine methylation and DDM1 in specifically maintaining transcriptional silencing of transposable elements, and indicates that other factors may contribute to the maintenance of transcriptional silencing for pseudogene sequences.

**Figure 2:**
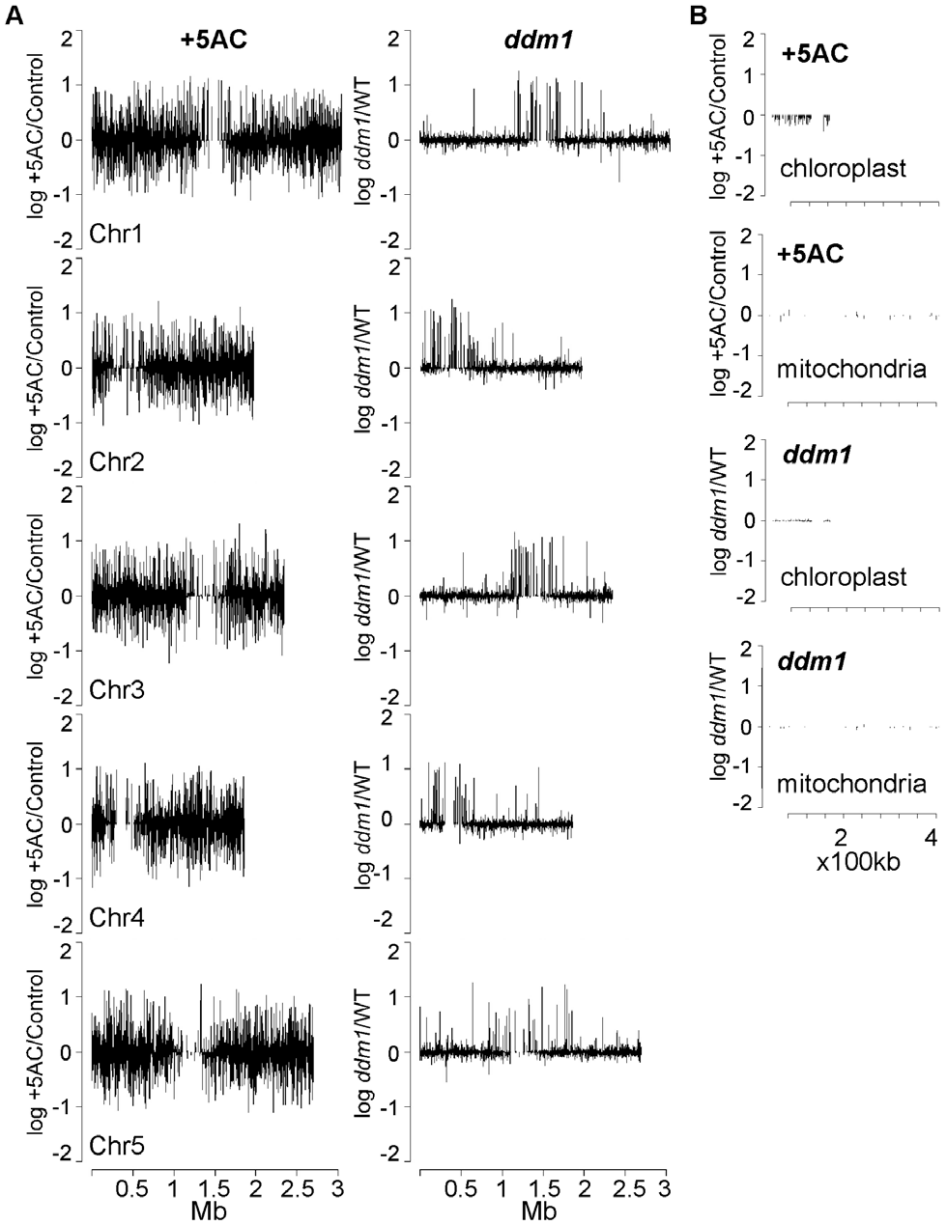
Genes misregulated in *ddm1* are not evenly distributed in the Arabidopsis genome. A. log_10_ fold-difference values (5-AC/Ws) and (*ddm1*/Ws) are plotted by transcription start site for 5 Arabidopsis chromosomes. B. log_10_ fold-difference values for 5-AC treated and *ddm1* seedlings for chloroplast and mitochondrial genes.

To determine if gene expression changes resulting from changes in DNA methylation and chromatin structure were randomly distributed throughout the genome, average expression change for all the genes called present on the array was plotted against the chromosomal position of the genes obtained from AGI annotations. Figure 2 shows that genes up- or down-regulated after 5-AC treatment appear to be distributed evenly throughout the genome, and change in expression by up to 100-fold. In contrast, in the *ddm1* mutant, relatively few genes changed dramatically in expression. The genes for which expression was altered were almost all up regulated, and these genes were located primarily in centromeric and pericentromeric regions. We also examined expression changes for genes encoded by subcellular organelle genomes (mitochondrion and chloroplast). We found that genes in the chloroplast were reduced in expression after 5-AC treatment (Figure 2B). Expression of genes in the mitochondrial genome (although there were fewer mitochondrial genes than chloroplast genes on the array) did not change significantly in response to 5-AC treatment, and the magnitude of change in expression levels of both plastid and mitochondrial genes was small in the *ddm1* mutant, as is expected from the nuclear localization of DDM1 and its role in nuclear chromatin remodeling.

We compared the genes identified as significantly differentially regulated in this study with genes identified in previously published studies which also examined the effects of chemically disrupted chromatin structure on gene expression in *Arabidopsis* [25]. We found that 24 of the 73 genes identified in a previous study which examined the effects of 5-AC on gene expression were upregulated in the present study, and 18 of the 52 genes previously identified as down-regulated by 5-AC were consistently downregulated in our experimental conditions [22]. Genes that were common to both experiments are listed in Table S4. The relatively small overlap in these two experiments may be explained by the use of different microarray platforms covering distinct sets of transcripts, as well as different experimental conditions. The Affymetrix 22K platform includes a number of genes that are not included on other microarrays, in particular genes located near the centromere and probes for transposable element genes. It is also likely that epigenetic changes induced by these chemical treatments are likely to vary significantly from cell to cell within an individual as well as between experiments.

### DNA damage response genes are upregulated in response to 5-AC treatment

**Figure 3:**
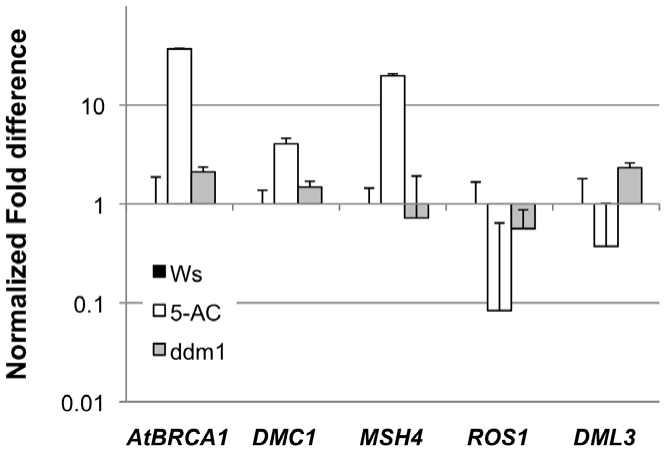
DNA damage repair genes are upregulated after 5-AC treatment. Quantitative RT-PCR expression ratios (5-AC and ddm1 normalized to Ws untreated control) for genes associated with repair of DNA damage or chromatin structure. Error bars indicate 95% confidence interval. *AtBRCA1, ARABIDOPSIS THALIANA BREAST CANCER SUSCEPTIBILITY1* (At4g21070); *DMC1, DISRUPTION OF MEIOTIC CONTROL1* (At3g22880); *ROS1, REPRESSOR OF SILENCING1* (At2g36490); *MSH4, MUTS HOMOLOG4* (At4g17380).

We observed that a number of the genes moderately but significantly upregulated in 5-AC treated seedlings, such as the *DMC1* and *AtBRCA1* genes had putative or documented roles in DNA repair. We found that these genes were also induced by bleomycin and mitomycin treatment as profiled in AtGenExpress genotoxic stress timecourse dataset (ExpressionSet_ME000326, http://www.arabidopsis.org/servlets/TairObject?type=expression_set&id=1007966782) (Figure S4). These genes were not differentially regulated in the *ddm1* mutant, and this suggests that 5-AC treatment may either result in increased DNA damage or double stranded breaks and that this DNA damage results from an aspect of chromatin remodeling not affected in the *ddm1* mutant. This is consistent with findings in other systems regarding the effects of 5-AC treatment [19], [23]. Several genes involved in DNA-damage repair were chosen for validation by quantitative reverse-transcriptase PCR (Figure 3). In contrast to other genes with a role in DNA repair, several genes for DNA base excision repair, including *REPRESSOR OF SILENCING1* (*ROS1*) and *DEMETER-LIKE3* (*DML3*), were significantly down-regulated in response to 5-AC treatment as well as in the *ddm1* mutant. *ROS1* and *DML3* encode DNA glycosylases that have been shown to be involved in active de-methylation of targets by base excision repair [24], [25], [26], this may indicate that these genes are negatively regulated by genomic hypomethylation or other effects of 5-AC treatment.

### Correlation with the other *Arabidopsis* chromatin mutants

The *homology dependent gene silencing1* (*hog1*) mutant is deficient in the *S*-adenosyl-L-homocysteine (SAH) hydrolase gene and shows reduced DNA methylation and upregulation of transposable element genes in genome wide expression profiling [27]. We compared expression of genes upregulated by *ddm1* in this experiment to the top 200 overexpressed genes in the *hog1* mutant (which used the same array as this study [28]) and observed that 26 of the *ddm1* overexpressed genes from our experiment were represented in the 200 most-overexpressed genes from the *hog1* mutant, and 83 of the genes significantly expressed after 5-AC treatment were also overexpressed in *hog1*. An independent study profiled gene expression in another *ddm1* allele and the *mom1* mutant [29]. Only 10 of the 35 genes upregulated in *mom1* and *ddm1* were found to be upregulated after 5-AC treatment in the present study (Table S5). None of the *ddm1*-upregulated genes from this study were found to be co-regulated by *ddm1* and *mom1* in the previous study [29]. The array platform used for the *ddm1 mom1* profiling did not contain probes to interrogate as many of the transposable element open reading frames as the microarray used in the present study, therefore an extensive direct comparison is not possible. The response of the *ddm1*-regulated genes from this experiment in these related studies is shown graphically in Figure S5.

The role of methylation in control of gene expression on a global scale has previously been determined for the *Arabidopsis met1* cytosine methyltransferase mutants lacking CG methylation, and the triple mutant *drm1drm2cmt3* (*ddc*) which lacks CNN/CNG methylation [30]. We compared the genes significantly overexpressed in the *met1* mutant identified in that study with the genes significantly up-regulated after treatment with 5-AC and in the *ddm1* mutant. The majority (243) of the 319 MET1-regulated genes correspond to transcripts that are not present on the ATH1 array, located around the centromeres and pericentromeres. Of the 76 remaining genes, 13 were below the threshold of detection for our experiment, but 27 (35%) of these genes were found to be significantly up-regulated after 5-AC treatment, a fraction which is significantly higher than would be expected of two random gene sets of this size (Table S5). 14 of these 27 genes are annotated as transposable element-related sequences. 8 of the genes upregulated in *met1* were found to be in the small and exclusive set of genes significantly upregulated in the *ddm1* mutant. In contrast, the genes that are up-regulated in the *drm1drm2cmt3* triple mutant (which are well-represented on the ATH1 microarray used in this study) are located in euchromatic regions, and only 15% are represented in the list of genes upregulated by 5-AC treatment. This fraction is close to what would be expected from comparing two random sets of genes this size (Table S5). None of these genes are significantly upregulated in the *ddm1* mutant. Taken together, these results are consistent with a model where the *ddm1* mutant is primarily affected in CG methylation, which is maintained by the MET1 methyltransferase and inhibited by 5-AC, and this form of cytosine methylation is necessary and sufficient for the transcriptional silencing of transposable elements located in centromeric and pericentromeric regions. CNG/CNN methylation requiring *DRM1*, *DRM2* and *CMT3*, which is not inhibited by 5-AC, affects a different subset of genes located in euchromatin which have a variety of developmental functions, and their expression is largely unaffected by the *ddm1* mutation (Figures S7 and S8) [30], [31].

## Discussion

These results indicate that use of inhibitors of cytosine methylation can directly or indirectly influence the expression of genes throughout the genome, but that the loss of DDM1 has little effect on the expression of genes that are influenced by DNA methylation but are located in the euchromatic chromosome arms. This finding is consistent with the reported role of DDM1 in maintaining heterochromatic silence of transposable elements, and the tendency for transposable elements to occur most frequently in the centromeric and pericentromeric regions [32], [33]. Other heterochromatin-remodeling proteins, for example, CHROMATIN ASSEMBLY FACTOR-1, do not exhibit this kind of regionally-delimited regulatory activity [34]. Many of the transposable-element encoding sequences are highly and significantly upregulated in the *hog1* mutant as well as in the *ddm1* mutant (Figure S5) [28]. This is consistent with the role of DNA methylation, requiring HOG1, DDM1, and MET1 in maintaining transcriptional silence of transposable elements [8].

The significant and unexpected effect on 5-AC expression on plastid gene expression may indicate that this chemical treatment could affect plastid metabolism or viability, possibly due to misexpression of vital nuclear encoded plastid proteins. Consistent with this observation, seedlings treated with 5-AC have a chlorotic, stunted appearance suggesting that plastid health may be compromised, in contrast to *ddm1* mutant seedlings that appear identical to wild-type seedlings at this stage (Figure S3.) Although a subset of the genes that changed in expression after 5-AC treatment were found to be misregulated in the chromatin mutants, indicating a role for methylation in regulation of expression (Table S5), the majority of genes which increased or decreased in expression were not found to be misregulated in the chromatin mutants (Figure S6). The global effects of 5-AC on gene expression may be a result of indirect effects of this inhibitor which result in DNA damage. Studies which include chemical treatments that perturb methylation are complicated by the fact that genomic hypomethylation caused by 5-AC treatment has also been shown to induce double stranded DNA breaks, and expression of DNA-damage response genes [19], [23]. Treatment conditions are clearly an important variable when using chemical inhibitors of chromatin structure. Analysis of gene expression in mutants with specific defects in chromatin organization, such as *ddm1*, reveal effects on transcription for genes in the transposable elements class consistent with changes in chromatin structure confined to heterochromatic regions.

## Materials and Methods

### Plant materials and growth conditions

Wild-type *Arabidopsis* (ecotype Ws) and *ddm1* seeds were germinated in 0.5× MS liquid media (containing 1% sucrose) at 21°C in continuous white light. For 5-AC treatments, 20 mg/L 5-AC (5-aza-2′deoxycytidine, Sigma #A-3656) in DMSO was added to media after germination, and media and inhibitors were replaced for all seedlings after 5 days, tissue was harvested after 14 days. The *ddm1* mutant used in this study was isolated from the Wisconsin T-DNA insert collection in the Ws ecotype by PCR screening. The T-DNA is located 2275 base pairs from the translation start site. The homozygous mutant was backcrossed and allowed to self-pollinate for 3 generations, and homozygous mutants were found to have reduced genomic DNA methylation (SL, unpublished results). RNA was extracted using the Qiagen Plant RNeasy kit according to manufacturers instructions (Qiagen, Valencia, CA, USA). Three biological replicates were performed for the wild-type, mutant, and treatment.

For verification of gene expression, seedlings were grown independently and RNA extracted as described above, and reverse transcription was performed on 5 µg RNA using the SuperScript III Kit (18080-051, Invitrogen, Carlsbad, CA) and this was diluted 10 fold for use as a template for quantitative PCR (qPCR). qPCR was performed on the ABI 7300 Sequence Detection System at the University of Chicago Functional Genomics Facility, with ABI SYBR-Green PCR core reagent kits (#4304886, Applied Biosystems, Foster City, CA). Transcript abundance was normalized to expression of the ACTIN7 gene, and expressed relative to wild type levels for three biological replicates. A list of qPCR primer sequences is provided in Table S6. For bisulfite sequencing, DNA was prepared as described [7] and treated using the EZ DNA Methylation-Gold Kit (Zymo Research, CA), a ∼500 bp methylated region of At1g65380 (*CLAVATA2*) was amplified for sequencing, captured in the pCR2.1TOPO cloning vector (Invitrogen, Carlsbad, CA) and ten clones were sequenced for each treatment/genotype. For chromatin immunopreciptiation, the Chromatin Immunoprecipitation Assay Kit (Millipore, 17–295) was used following kit protocols, and DNA was precipitated using the anti 5-methylcytosine polyclonal antibody (20-CS9, Fitzgerald Industries International, Concord, MA).

### Microarray Data Analysis

The array used was the Affymetrix ATH1 array with approximately 22,000 genic probesets. Microarray expression was calculated using gcRMA 2.2.0 as implemented in Bioconductor 1.7 [35]. We first selected genes (17204 probesets) with a “present” call on 2 or more arrays using Affymetrix MAS 5.0 software. To identify differentially expressed genes we used a two-tailed, type 3, t-test followed by false discovery rate correction for multiple testing with a significance cutoff of  = 0.05 [36], as implemented in the Bioconductor GeneTS package). Gene annotations were obtained from the AGI annotation of Affymetrix array elements, available at (ftp://ftp.arabidopsis.org, dated 7-29-2009). Positions of genes were obtained from the AGI annotation of Affymetrix array elements, available at (ftp://ftp.arabidopsis.org, dated 7-14-2006), and corresponding to the closest match for the probeset. Plots of gene expression on the chromosomes were generated in R v. 2.2.0. MIAME-compliant microarray data from this study is deposited in the Gene Expression Omnibus (http://www.ncbi.nlm.nih.gov/geo) as accession number GSE25067. Gene expression data for other chromatin mutants used for purposes of comparison was obtained from publicly available datasets at GEO (GSE6166, GSE5771, and GSE5074).

## Supporting Information

Figure S1 **Cytosine methylation after 5-AC treatment and in the** ***ddm1*** **mutant.** A. Quantitation of C, CNG, and CG methylation determined by bisulfite sequencing of a methylated region of the *CLAVATA2* gene promoter to assess cytosine methylation in control, 5-AC-treated, and *ddm1* seedlings, expressed as a percentage of the total number of cytosine residues in the sequence. B. Chromatin immunoprecipitation from control and 5-AC-treated DNA was followed by PCR amplification of centromeric repeats to assess cytosine methylation. Leftmost lane on top and bottom is the DNA size marker, primer pair 3 amplifies 180 bp-centromeric repeats, which are methylated in control DNA. Precipitation efficiency is reduced in 5-AC-treated DNA. The other primer sets (lanes 1,2, and 4) are part of an independent study.
(TIF)

Figure S2 **Fold difference and p-values for microarray experiment.** *p*-values and log_2_ fold-change values from microarray data. A. For 5-AC treated seedlings. B. For *ddm1* mutant seedlings. False discovery-corrected significance cutoff for each experiment is indicated by the red line.
(TIF)

Figure S3 **5-AC treated seedlings.** Control (Ws) and 5-AC treated seedlings after 14 days growth in experimental conditions (see methods).
(TIF)

Figure S4 **5-AC response is correlated with genotoxic response.** Comparison of induction of selected 5-AC responsive genes with putative functions in DNA damage repair and after treatment with mitomycin and bleomycin (genotoxic stress timecourse dataset from AtGenExpress).
(TIF)

Figure S5 **Expression of** ***ddm1*** **-upregulated genes in other expression profiling studies.** Log fold difference for mutant/wild-type or treatment/control for 121 genes with the largest *ddm1-101*/control fold change (from this study) visualized by hierarchical clustering. Missing values (genes not interrogated by the array) for the *mom1* and *ddm1-5* samples [29] are in white.
(TIF)

Figure S6 **Expression of genes affected by 5-AC treatment in other expression profiling studies.** Log fold difference for mutant/wild-type or treatment/control for 3347 genes with the largest 5-AC/control fold change (from this study) visualized by hierarchical clustering. Missing values (genes not interrogated by the array) for the *mom1* and *ddm1-5* samples [29] are in white.
(TIF)

Figure S7 **Expression of** ***met1*** **-regulated genes in** ***ddm1*** **and 5-AC treated seedlings.** Log fold difference for mutant/wild-type or treatment/control for 200 genes found to be upregulated in the *met1* mutant [30]. Missing values for the *ddm1* and 5-AC samples are in white.
(TIF)

Figure S8 **Expression of** ***ddc*** **-regulated genes in** ***ddm1*** **and 5-AC treated seedlings.** Log fold difference for mutant/wild-type or treatment/control for 213 genes found to be upregulated in the *ddc* triple mutant [30]. Missing values for the *ddm1* and 5-AC samples are in white.
(TIF)

Table S1 Genes significantly up- or down-regulated in the *ddm1* mutant.
(XLSX)

Table S2 Genes significantly upregulated after 5-AC treatment.
(XLSX)

Table S3 Genes significantly downregulated after 5-AC treatment.
(XLSX)

Table S4 Genes with common regulation between 5-AC treatment experiments.
(XLSX)

Table S5 Overlap between genes upregulated in *ddm1* or after 5-AC and other global gene expression studies in chromatin mutants.
(XLSX)

Table S6 Primer sequences for qPCR.
(XLSX)
