# Supplementary figures and images for: Changes in Global Gene Expression in Response to Chemical and Genetic Perturbation of Chromatin Structure
Source: PLoS One. 2011 Jun 3;6(6):e20587. doi: 10.1371/journal.pone.0020587 (PMC3108824; doi:10.1371/journal.pone.0020587)

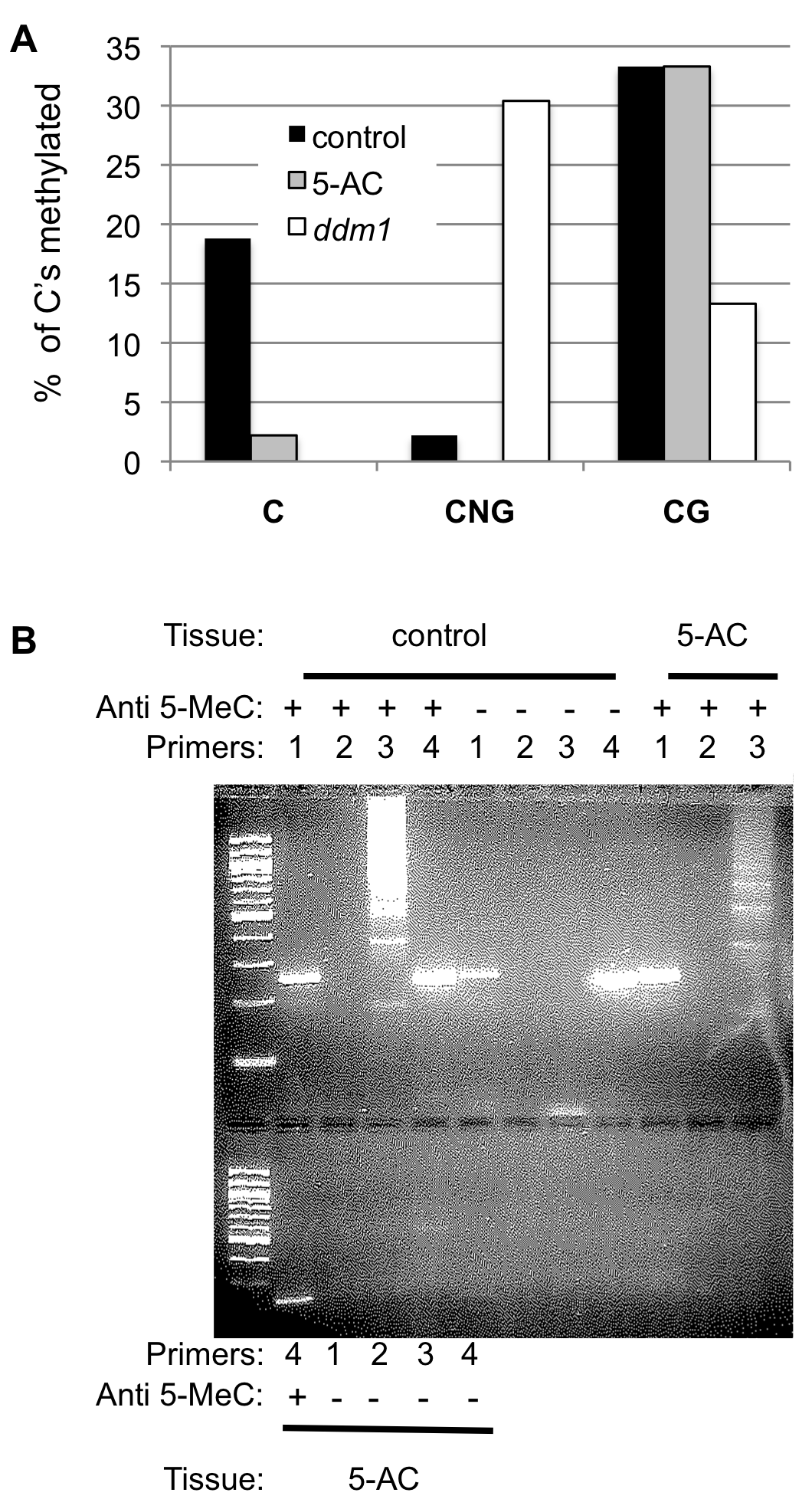

Supplement: Figure S1 — Cytosine methylation after 5-AC treatment and in the ddm1 mutant. A. Quantitation of C, CNG, and CG methylation determined by bisulfite sequencing of a methylated region of the CLAVATA2 gene promoter to assess cytosine methylation in control, 5-AC-treated, and ddm1 seedlings, expressed as a percentage of the total number of cytosine residues in the sequence. B. Chromatin immunoprecipitation from control and 5-AC-treated DNA was followed by PCR amplification of centromeric repeats to assess cytosine methylation. Leftmost lane on top and bottom is the DNA size marker, primer pair 3 amplifies 180 bp-centromeric repeats, which are methylated in control DNA. Precipitation efficiency is reduced in 5-AC-treated DNA. The other primer sets (lanes 1,2, and 4) are part of an independent study. (TIF) [file pone.0020587.s001.tif]

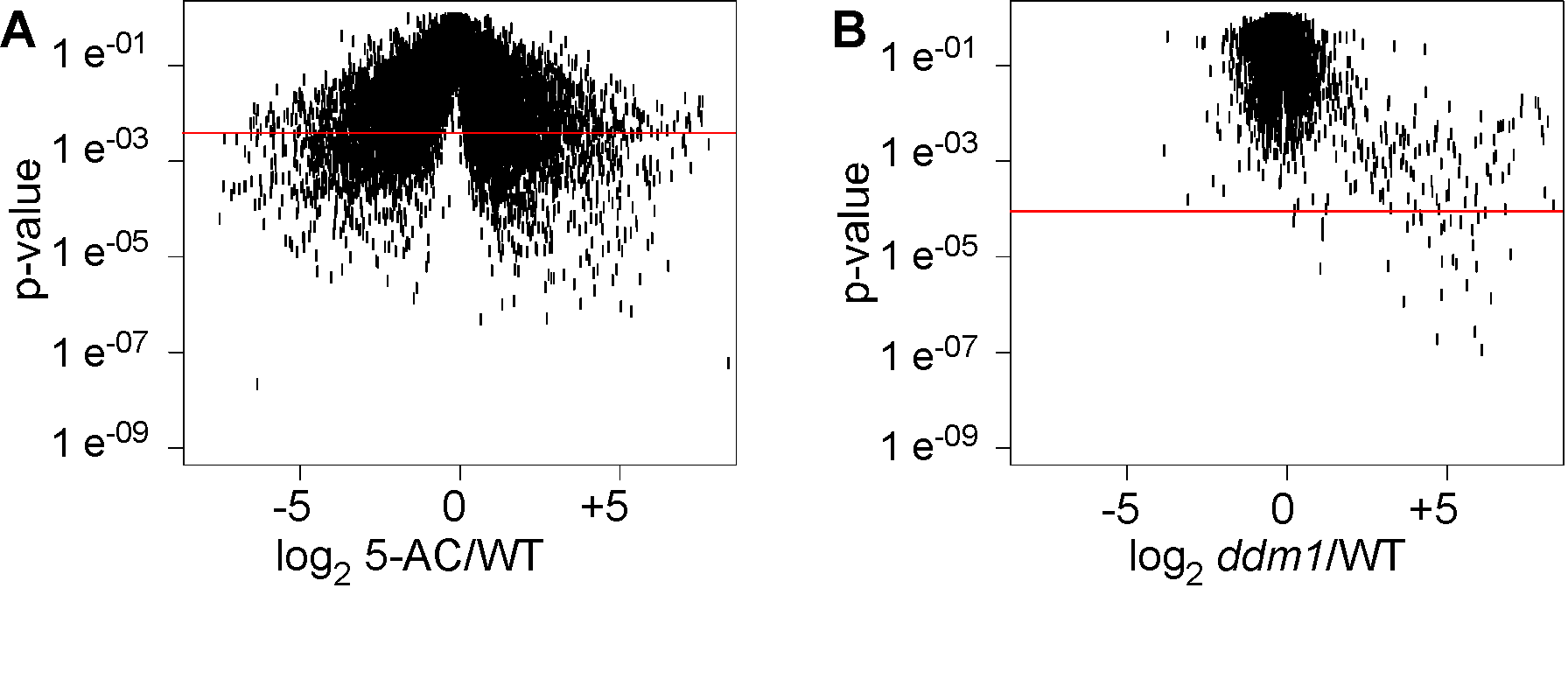

Supplement: Figure S2 — Fold difference and p-values for microarray experiment. p-values and log2 fold-change values from microarray data. A. For 5-AC treated seedlings. B. For ddm1 mutant seedlings. False discovery-corrected significance cutoff for each experiment is indicated by the red line. (TIF) [file pone.0020587.s002.tif]

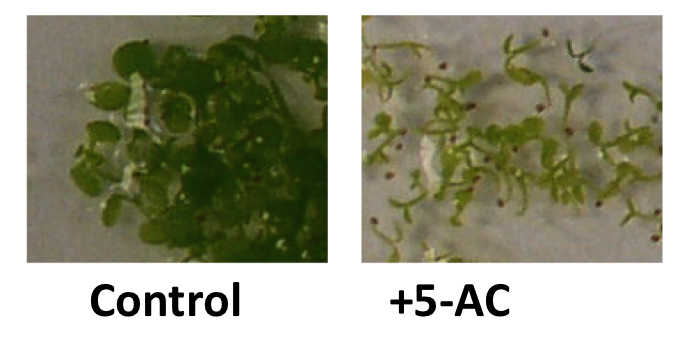

Supplement: Figure S3 — 5-AC treated seedlings. Control (Ws) and 5-AC treated seedlings after 14 days growth in experimental conditions (see methods). (TIF) [file pone.0020587.s003.tif]

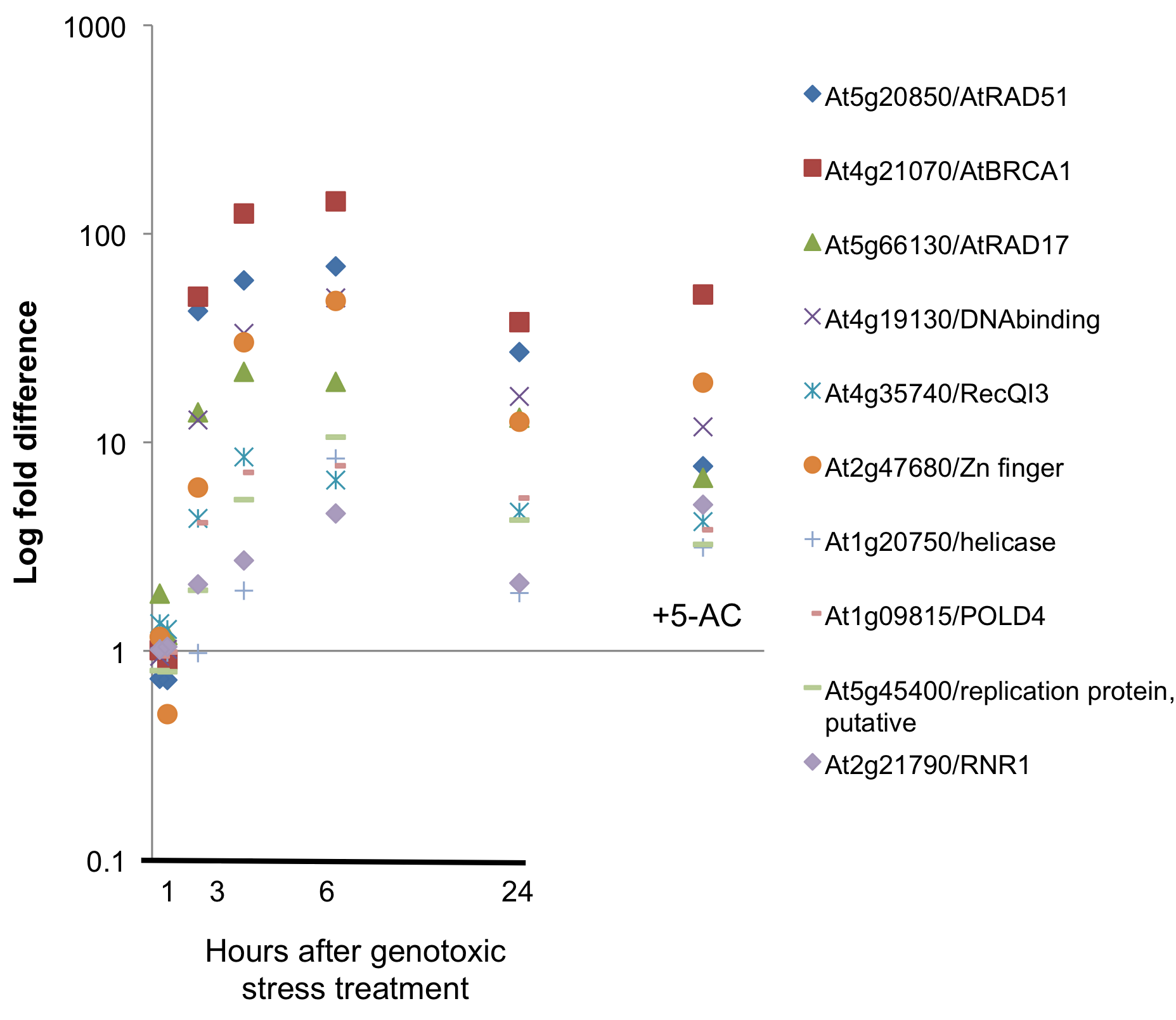

Supplement: Figure S4 — 5-AC response is correlated with genotoxic response. Comparison of induction of selected 5-AC responsive genes with putative functions in DNA damage repair and after treatment with mitomycin and bleomycin (genotoxic stress timecourse dataset from AtGenExpress). (TIF) [file pone.0020587.s004.tif]

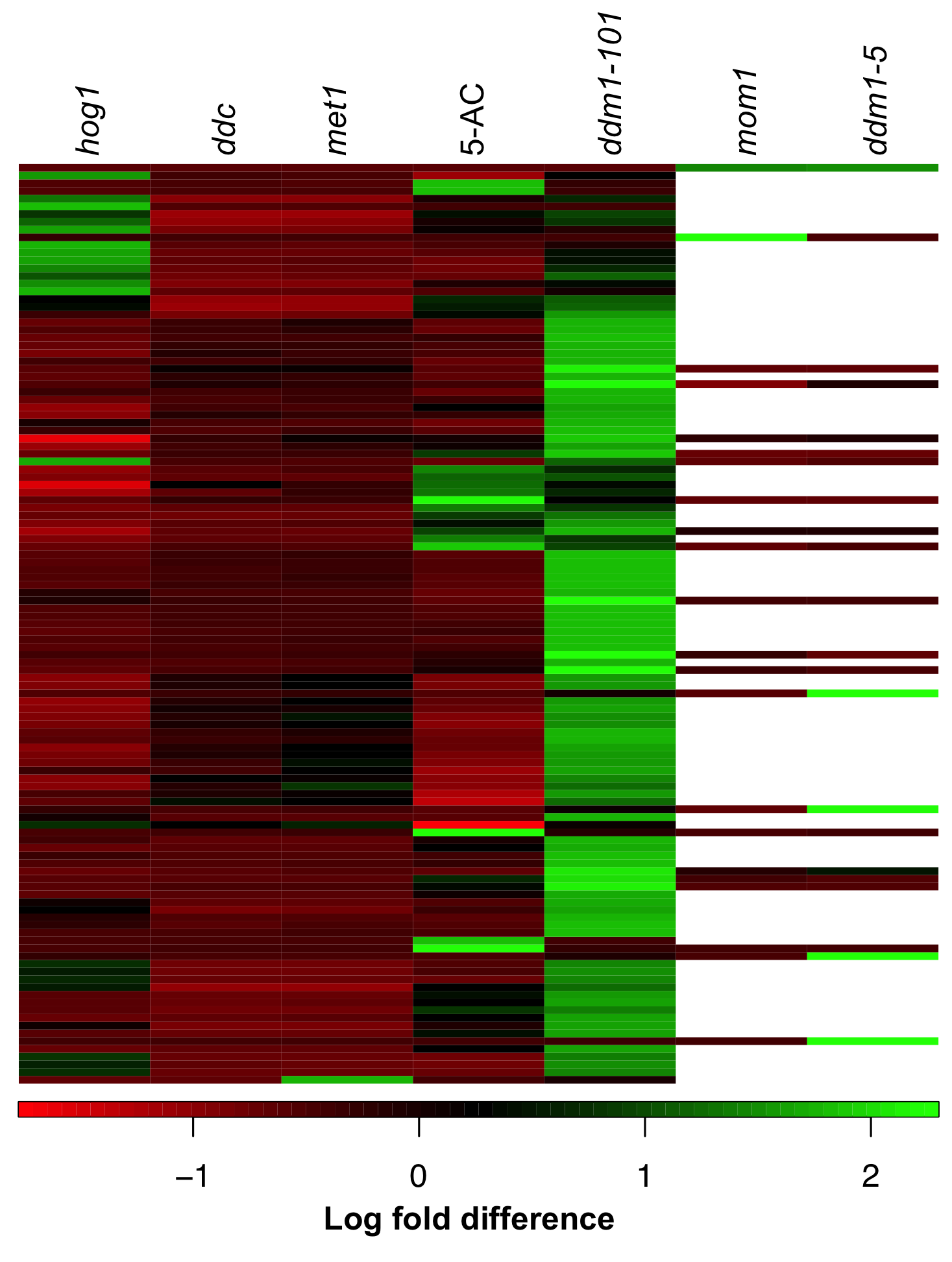

Supplement: Figure S5 — Expression of ddm1 -upregulated genes in other expression profiling studies. Log fold difference for mutant/wild-type or treatment/control for 121 genes with the largest ddm1-101/control fold change (from this study) visualized by hierarchical clustering. Missing values (genes not interrogated by the array) for the mom1 and ddm1-5 samples [29] are in white. (TIF) [file pone.0020587.s005.tif]

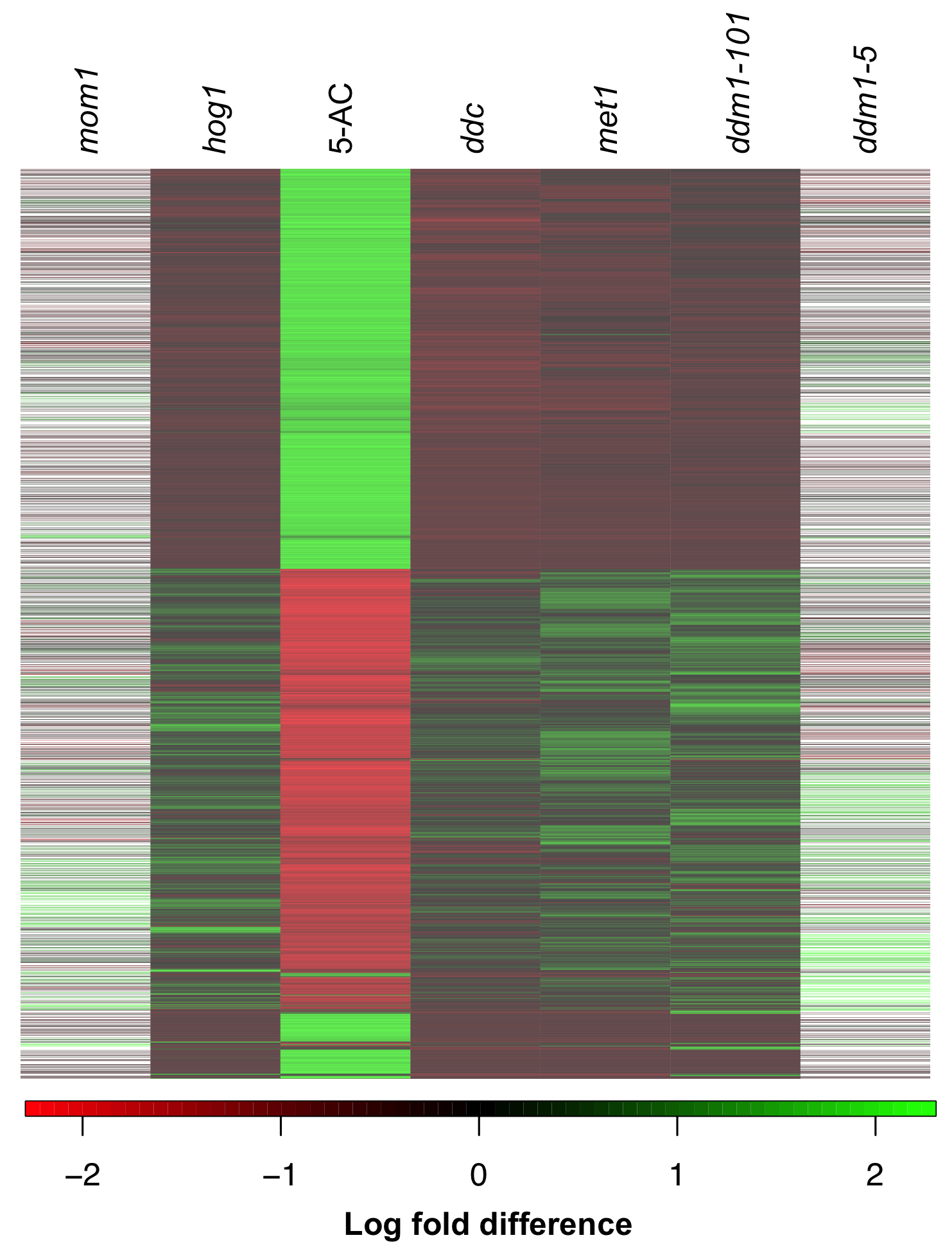

Supplement: Figure S6 — Expression of genes affected by 5-AC treatment in other expression profiling studies. Log fold difference for mutant/wild-type or treatment/control for 3347 genes with the largest 5-AC/control fold change (from this study) visualized by hierarchical clustering. Missing values (genes not interrogated by the array) for the mom1 and ddm1-5 samples [29] are in white. (TIF) [file pone.0020587.s006.tif]

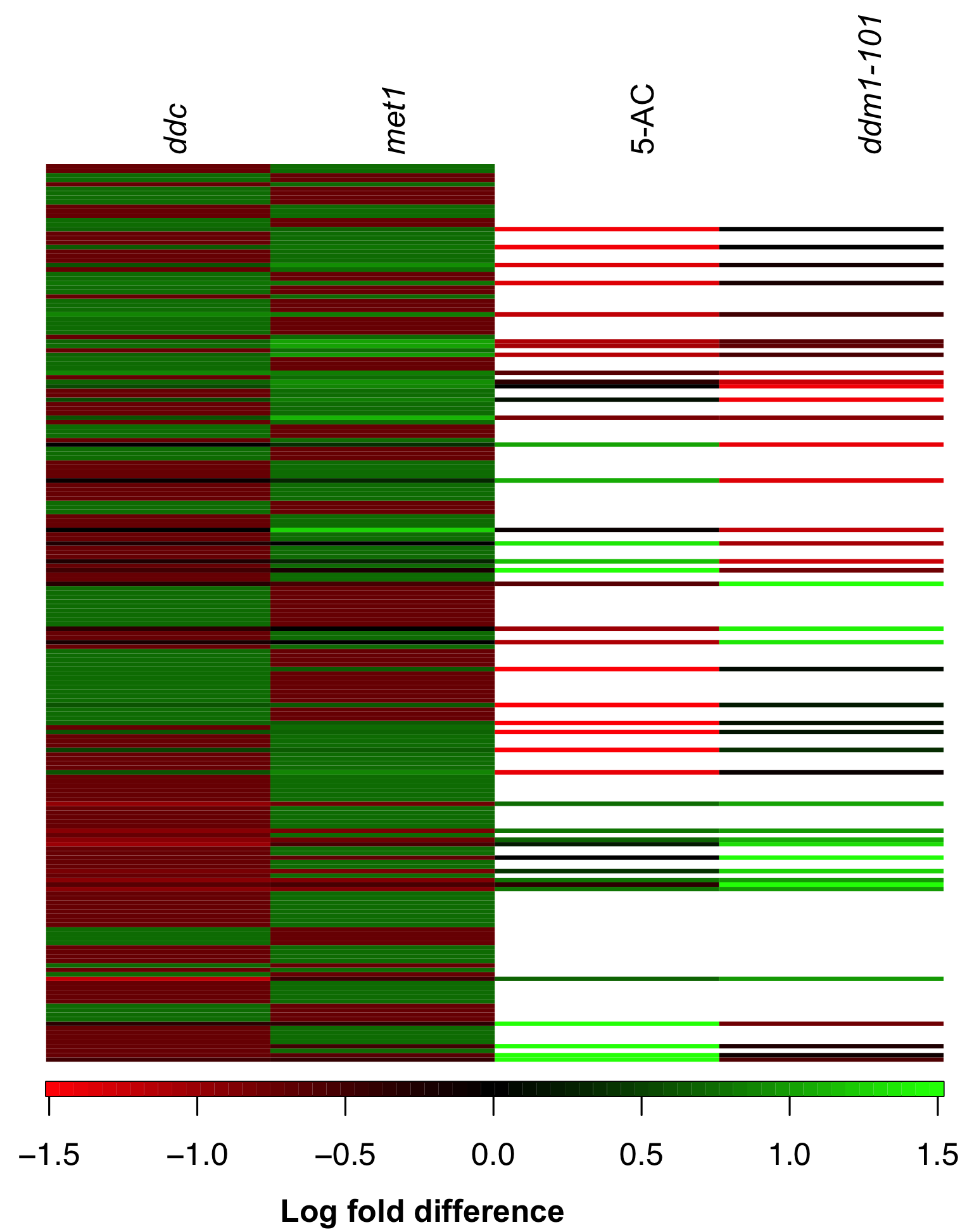

Supplement: Figure S7 — Expression of met1 -regulated genes in ddm1 and 5-AC treated seedlings. Log fold difference for mutant/wild-type or treatment/control for 200 genes found to be upregulated in the met1 mutant [30]. Missing values for the ddm1 and 5-AC samples are in white. (TIF) [file pone.0020587.s007.tif]

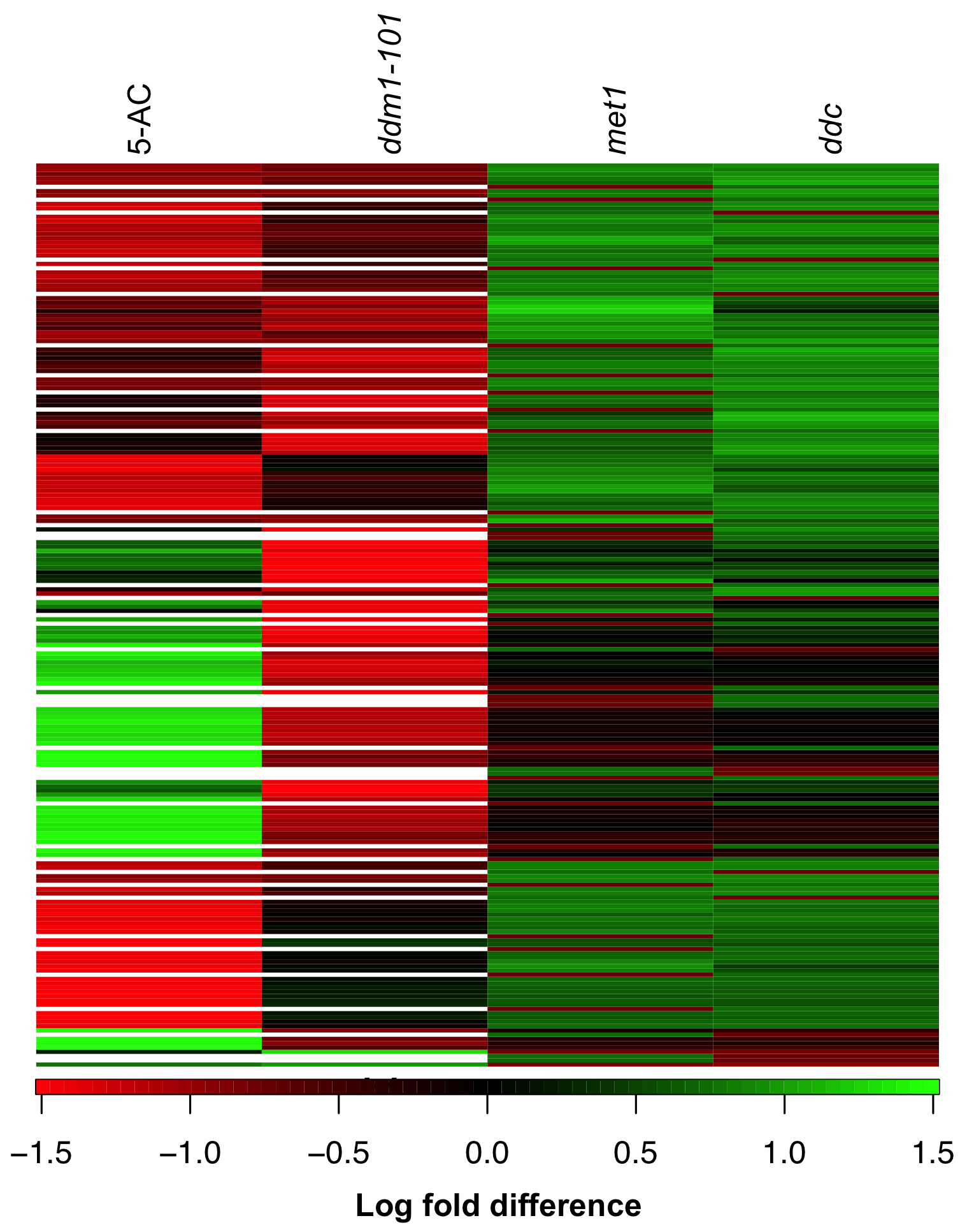

Supplement: Figure S8 — Expression of ddc -regulated genes in ddm1 and 5-AC treated seedlings. Log fold difference for mutant/wild-type or treatment/control for 213 genes found to be upregulated in the ddc triple mutant [30]. Missing values for the ddm1 and 5-AC samples are in white. (TIF) [file pone.0020587.s008.tif]
